# bZIP-Domain Variant Allele Frequency Helps to Refine Risk Stratification in CEBPA-Mutated AML

**DOI:** 10.3390/biomedicines14010256

**Published:** 2026-01-22

**Authors:** Kainan Zhang, Xiaohang Ma, Xiaoxuan Lu, Guorui Ruan, Fangfang Wei, Hao Jiang, Yingjun Chang, Xiaojun Huang, Xiaosu Zhao

**Affiliations:** 1 National Clinical Research Center for Hematologic Disease, Beijing Key Laboratory of Hematopoietic Stem Cell Transplantation, Peking University Institute of Hematology, Peking University People’s Hospital, Beijing 100044, China; 2311110390@bjmu.edu.cn (K.Z.);; 2 Peking-Tsinghua Center for Life Sciences, Academy for Advanced Interdisciplinary Studies, Peking University, Beijing 100871, China; 3 Research Unit of Key Technique for Diagnosis and Treatments of Hematologic Malignancies, Chinese Academy of Medical Sciences, Beijing 100730, China; 4 Collaborative Innovation Center of Hematology, Peking University, Beijing 100044, China

**Keywords:** acute myeloid leukemia, CEBPA, prognosis

## Abstract

**Objectives**: To investigate the prognostic value of CEBPA (CCAAT/enhancer-binding protein α) molecular features, such as variant allele frequency (VAF), in patients with de novo acute myeloid leukemia (AML). **Methods**: Next-generation sequencing (NGS) was used to detect CEBPA mutations in 162 patients with newly diagnosed AML (except acute promyelocytic leukemia). **Results**: We established 44.2% as the optimal threshold for both maximum VAF and bZIP-domain VAF. The high-VAF group showed higher leukemia burden and inferior event-free survival (EFS). bZIP-domain VAF demonstrated superior prognostic value over maximum VAF (HR: 3.174 vs. 2.460) and was validated across subgroups, namely cytogenetically normal acute myeloid leukemia (CN-AML), chemotherapy-only, and low/intermediate-risk subgroups. Multivariate analysis confirmed high bZIP-domain VAF and DNMT3A mutation as independent risk factors. **Conclusions**: Our results confirm that the bZIP-domain VAF of CEBPA mutations is a more effective predictor of relapse than the maximum VAF, offering a valuable tool for the early identification of patients at high risk of relapse.

## 1. Introduction

Acute myeloid leukemia (AML) is a highly heterogeneous malignant hematologic disease, accounting for approximately 80% of adult acute leukemias. Its molecular genetic characteristics play a critical role in disease diagnosis, risk stratification, treatment selection, and prognosis assessment [1,2]. The CEBPA (CCAAT/enhancer-binding protein α) gene, located on chromosome 19, encodes a protein containing an N-terminal transactivation domain (TAD) and a C-terminal basic region leucine zipper (bZIP) domain. The protein primarily binds DNA via the bZIP domain, with the N-terminal TAD mediating transactivation, thereby regulating gene transcription and playing a vital role in modulating cell differentiation and glucose metabolism [3,4,5]. CEBPA mutations are relatively common in AML, with an incidence of approximately 7–11% [6]. Its mutation sites, variant allele frequency (VAF), copy number variations (CNVs), and the occurrence of germline mutations vary among patients. Based on the mutation site, CEBPA mutations can be classified into those affecting the C-terminal bZIP domain, the N-terminal TAD domain, and biallelic mutations involving both termini. The WHO 2022 classification [7] defines AML with biallelic CEBPA mutations and monoallelic bZIP-domain mutations as a distinct subtype, whereas the ELN 2022 [8] and ICC guidelines [9] consider in-frame CEBPA mutations as a favorable prognostic category based on recent studies [10,11], suggesting that the prognostic value of CEBPA mutation sites remains uncertain. Conversely, studies investigating the prognostic impact of the variant allele frequency (VAF) of CEBPA mutations are relatively limited, often restricted to specific patient subgroups, and have generated conflicting results. One study involving 15 patients with monoallelic CEBPA mutations suggested that a VAF ≥ 30% was associated with longer overall survival (OS) [12]. Another study of 141 patients with cytogenetically normal AML (CN-AML) harboring CEBPA bZIP mutations found that a VAF > 45.45% and indels involving ≥3 base pairs were associated with a higher cumulative incidence of relapse (CIR) [13]. Therefore, the utility of various CEBPA mutational characteristics for prognostic stratification warrants further investigation. This study aims to explore the relationship between the molecular features of CEBPA mutations and event-free survival (EFS) in a large single-center Chinese patient cohort, analyzing the prognostic value of these molecular characteristics.

## 2. Materials and Methods

### 2.1. Participants of This Study

**Figure 1:**
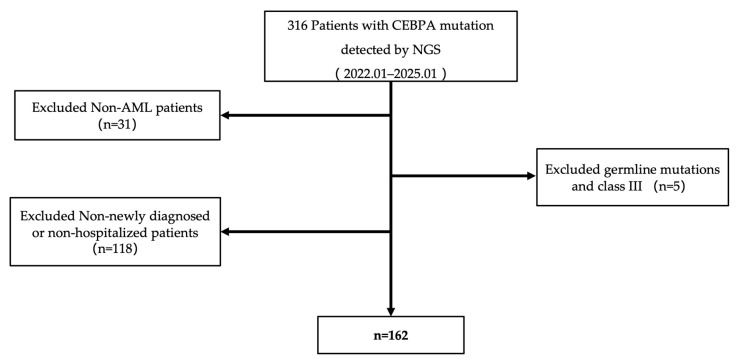
Patient enrollment flowchart.

The data for this study were retrospectively collected from the Department of Hematology, Peking University People’s Hospital. From January 2022 to January 2025, 316 patients with CEBPA mutations identified by next-generation sequencing (NGS) were initially enrolled from our institute. After excluding 31 non-AML patients, 118 AML patients who were not newly diagnosed or did not receive systematic chemotherapy at our institute, and 5 AML patients with germline or Class III CEBPA mutations, a final cohort of 162 newly diagnosed AML patients (excluding acute promyelocytic leukemia) was available for analysis (Figure 1). The patients’ clinical data were derived from the hospital’s medical record system, encompassing basic demographic information and various laboratory test results. All laboratory tests were performed at the time of initial diagnosis, prior to the initiation of any treatment. The cohort included 83 males and 79 females, with a median age of 43.5 years (5.0–73.0 years) and a median follow-up time of 9.5 months (1.4–58.0 months). All patients were diagnosed with AML based on MICM criteria, incorporating French–American–British (FAB) morphological classification and immunophenotyping by flow cytometry. Risk stratification was performed according to the 2022 ELN guidelines [8]. Among 162 patients, 45 received reduced-intensity chemotherapy (Venetoclax plus azacitidine). The follow-up period ended in July 2025. This study was approved by the Ethics Committee of Peking University People’s Hospital. All patients provided written informed consent to participate in the present study in accordance with the Declaration of Helsinki.

### 2.2. Mutation Detection

Next-generation sequencing (NGS) was used to detect the molecular characteristics of CEBPA, including mutation sites, variant allele frequency (VAF), copy number variations (CNV), and germline mutations. Genomic DNA was extracted from mononuclear cells isolated from bone marrow aspirates collected at initial diagnosis with a QIAsymphony SP nucleic acid extraction and purification analyzer (Qiagen, Hilden, Germany, 9001545). In this experiment, we strictly followed the manufacturer’s standard operating procedures, employing its dedicated nucleic acid extraction kit (QIAamp DNA Mini Kit) to complete DNA extraction for all samples to ensure purity and fragment length suitable for library preparation and high-throughput sequencing. The purified DNA was quantified and quality-controlled using a NanoDrop™ ND-2000C spectrophotometer (Thermo Fisher Scientific, Waltham, MA, USA). Sequencing libraries were prepared using the KAPA HyperPrep Kit (Roche Sequencing Solutions, Pleasanton, CA, USA) following a comprehensive hybridization capture workflow. Target enrichment was performed using the SureSelectXT Hybridization Capture Kit (Agilent Technologies, Santa Clara, CA, USA) with a 139-gene myeloid malignancy panel (Table S1). Hybridization was conducted at 65 °C for 16 h. Post-hybridization, the biotinylated probe-target DNA complexes were captured using Dynabeads MyOne Streptavidin T1 beads (Thermo Fisher Scientific, Waltham, MA, USA). Finally, the captured target DNA was eluted, purified, and amplified. Following library construction, paired-end sequencing (PE150) was conducted on the Illumina NovaSeq 6000 sequencing system (Illumina, San Diego, CA, USA). Sequencing was performed using an S4 flow cell to achieve high-output data generation. The average sequencing depth was >2000× to ensure sensitivity for low-level variants. The overall analytical sensitivity of the assay is approximately 1% variant allele frequency (VAF), which aligns with clinical standards for mutation detection.

Raw data were demultiplexed using bcl2fastq (v2.20). Reads were aligned to the human reference genome (GRCh37/hg19) using BWA-MEM (v0.7.17). Somatic variant calling for SNVs/InDels was performed with GATK Mutect2 (v4.2.0.0) and VarScan2 (v2.4.4), with a reporting threshold of VAF ≥ 1%. Copy number variations were analyzed using depth-based algorithms (e.g., CONTRA). Variants were annotated with ANNOVAR (v2020.06.07) and filtered against public databases (gnomAD, dbSNP, COSMIC). Each sequencing run included positive and negative controls.

The VAF was calculated according to the following formula: Variant Allele Frequency (VAF, %) = (Number of sequencing reads containing the mutant allele/Total number of sequencing reads [mutant + wild-type]) × 100%. In the analysis using maximum VAF, the maximum VAF was applied to all patients. For the analysis based on the bZIP-domain VAF, the bZIP-domain VAF was used where applicable, with the maximum VAF substituted for patients having only a monoallelic mutation or no bZIP-domain mutation. In patients harboring two mutations within the bZIP domain, the mutation with the highest VAF was chosen for further investigation.

### 2.3. Treatment and Assessment Method

Patients with hyperleukocytosis received pretreatment with hydroxyurea alone prior to induction chemotherapy. Induction regimens consisted of one to two cycles of IA (idarubicin 8–10 mg/m^2^ × 3 days, cytarabine 100 mg/m^2^ × 7 days), HAA (homoharringtonine 2 mg/m^2^ × 7 days, aclarubicin 20 mg/day × 7 days, cytarabine 100 mg/m^2^ × 7 days), MA (mitoxantrone 6 mg/m^2^ × 3 days, cytarabine 100 mg/m^2^ × 7 days), DA (daunorubicin 60 mg/m^2^ × 3 days, cytarabine 100 mg/m^2^ × 7 days), or CAG (aclarubicin 20 mg × 4 days, cytarabine 10 mg/m^2^ q12h × 7–14 days, recombinant human granulocyte colony-stimulating factor 300 μg × 7–14 days) regimens. Decitabine (20 mg/m^2^ × 5 days) was added based on peripheral white blood cell counts. Patients intolerant to intensive chemotherapy or elderly patients received venetoclax plus azacitidine. All patients who achieved remission received consolidation therapy comprising at least six cycles, including four cycles of intermediate-dose cytarabine (2 g/m^2^ q12h × 3 days) and two or more cycles of anthracycline-based regimens (daunorubicin 45 mg/m^2^ × 3 days, or idarubicin 10 mg/m^2^ × 3 days, or mitoxantrone 8 mg/m^2^ × 3 days) combined with cytarabine (100 mg/m^2^ × 7 days). Allogeneic hematopoietic stem cell transplantation (HSCT) was performed in 24 patients after the first complete remission and in 17 patients following re-induction after relapse.

Treatment response was evaluated according to the NCCN-AML Guidelines [14]. Complete remission (CR) was defined as an absolute neutrophil count >1 × 10^9^/L in peripheral blood, platelet count ≥100 × 10^9^/L, absence of extramedullary disease, and a bone marrow blast percentage of <5%. Relapse was considered upon the recurrence of leukemia cells in the peripheral blood, a bone marrow blast percentage ≥ 5%, the appearance of new dysplasia, or evidence of extramedullary disease in patients previously in CR. Event-free survival (EFS) was defined as the time from the date of diagnosis to disease relapse or death. CN-AML (cytogenetically normal acute myeloid leukemia) is defined as AML with no recurrent chromosomal abnormalities (e.g., t(8;21), inv(16), t(15;17), etc.) detected by conventional karyotyping (G-banding).

### 2.4. Statistical Analysis

Statistical analysis was performed using SPSS 26.0 (IBM Inc., Armonk, NY, USA). The Mann–Whitney U test was used for comparing two sets of data, while the chi-square test was employed for comparing proportions between two groups. Kaplan–Meier analysis was conducted to compare EFS between groups. Cox regression analysis was performed for both univariate and multivariate analyses. A *p*-value of <0.05 was considered statistically significant.

## 3. Results

### 3.1. Clinical Features of Patients with Different CEBPA Molecular Characteristics

**Figure 2:**
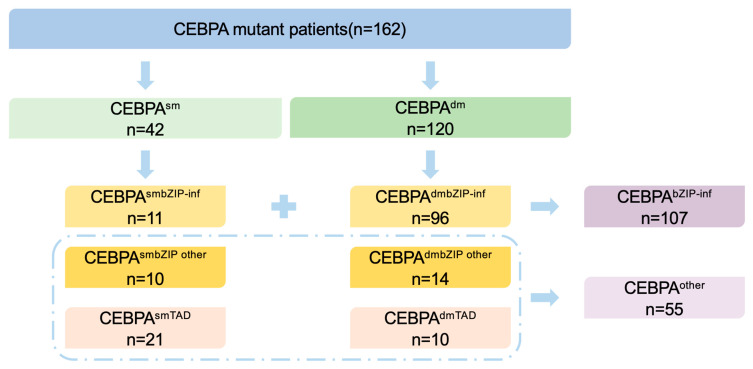
Grouping strategy of CEBPA-mutated patients by mutation sites. (The plus symbol indicates that CEBPA^smbZIP-inf^ and CEBPA^dmbZIP-inf^ together form the CEBPA^bZIP-inf^ group. The dashed line indicates that CEBPA^smbZIP-other^, CEBPA^dmbZIP-other^, CEBPA^smTAD^, and CEBPA^dmTAD^ together form the CEBPA^other^ group).

According to the 2022 ELN guidelines, we stratified patients into a CEBPA^bZIP-inf^ group (n = 107) and a CEBPA^Other^ group (n = 55) (Figure 2). The groups demonstrated significant differences in baseline characteristics (Table 1). Notably, the CEBPA^bZIP-inf^ group was significantly younger (39.0 years vs. 55.0 years, *p* < 0.0001) and exhibited higher hemoglobin levels (99.0 g/L vs. 80.0 g/L, *p* = 0.0001) yet lower platelet counts than the CEBPA^Other^ group (29.0 × 10^9^/L vs. 58.0 × 10^9^/L, *p* = 0.0027).

To investigate the impact of the variant allele frequency (VAF) of CEBPA mutations on clinical characteristics and patient prognosis, the maximum VAF and the bZIP-domain VAF were selected, respectively, as representative values for analysis in cases with biallelic mutations. The optimal cutoff value for VAF was determined using the time-dependent ROC curve analysis. Both methods identified a consistent optimal threshold of 44.2%, with comparable AUC values (0.571 vs. 0.574) (Figure S1). To validate the robustness of this threshold, we performed 5000 bootstrap resamples. The results showed that for both maximum VAF and bZIP-domain VAF, the median value from 5000 resamples remained 44.2%, and the median *p*-values were statistically significant (maximum VAF: *p* = 0.049; bZIP-domain VAF: *p* = 0.017), indicating that the identified VAF threshold is stable and reliable (Figures S2 and S3). When patients were stratified according to maximum VAF, the high-VAF group (>44.2%) exhibited a higher proportion of male patients (63.5% vs. 45.5%, *p* = 0.0413), a higher percentage of bone marrow blasts (67.3% vs. 55.0%, *p* = 0.0091), higher white blood cell counts (44.9 × 10^9^/L vs. 9.0 × 10^9^/L, *p* < 0.0001), and lower platelet counts (26.0 × 10^9^/L vs. 39.0 × 10^9^/L, *p* = 0.0050) compared to the low-VAF group (Table 2).

Similarly, stratification based on the bZIP-domain VAF revealed that the high-VAF group also had a significantly higher percentage of bone marrow blasts (72.8% vs. 54.5%, *p* < 0.0001) and higher white blood cell counts (44.9 × 10^9^/L vs. 9.7 × 10^9^/L, *p* < 0.0001) (Table 3).

### 3.2. Analysis of CEBPA Mutation Characteristics and Co-Mutation

**Figure 3:**
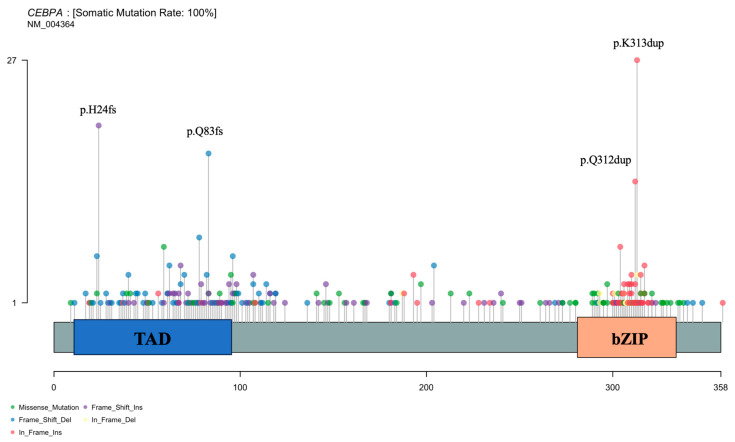
Landscape of CEBPA mutation sites. (Hotspots with higher frequencies are indicated in the figure.)

Mutation site distribution analysis of the 162 CEBPA-mutated patients (Figure 3) revealed that C-terminal bZIP domain mutations were predominantly in-frame mutations, with hotspots at K313dup and Q312dup. In contrast, N-terminal TAD domain mutations were primarily frameshift or missense mutations, with hotspots at H24fs and Q83fs.

Copy number variation (CNV) analysis was performed in 155 patients. The most frequent CNV was copy-neutral loss of heterozygosity (CN-LOH) on 11p involving WT1 (n = 26), which showed no significant impact on EFS (*p* = 0.17, Figure S4a). 13 patients (8.3%) carried CEBPA-associated 19q CN-LOH. Most of these patients (10/13, 77.0%) harbored CEBPA^bZIP-inf^ mutations with high CEBPA mutation VAF (median: 82.0%); however, 19q CN-LOH also did not affect EFS (*p* = 0.73, Figure S4b). Germline CEBPA mutations were identified in 3 of 61 tested patients (4.8%), all located in the N-terminal region with a median VAF of 45.9%.

**Figure 4:**
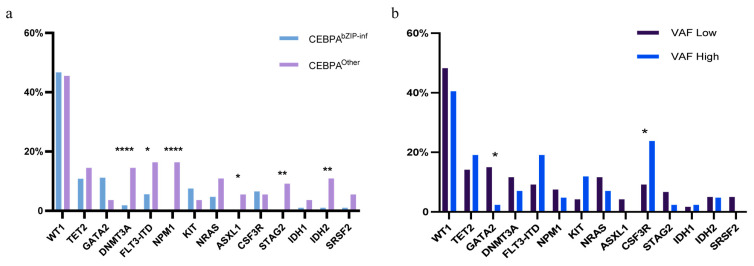
Co-mutation landscape in CEBPA-mutated AML: (**a**) Co-mutation profile stratified by CEBPA mutation type (CEBPA^bZIP-inf^ vs. CEBPA^Other^). (**b**) Co-mutation profile stratified by bZIP-domain VAF status (High vs. Low). (*p* < 0.05 *, *p* < 0.01 **, *p* < 0.0001 ****).

Analysis of cooperating mutations identified the most frequent co-mutations as WT1 (75/162, 46.3%), TET2 (25/162, 15.4%), GATA2 (19/162, 11.7%), FLT3-ITD (10/162, 6.2%), NRAS (11/162, 6.8%), DNMT3A (10/162, 6.2%), and KIT (10/162, 6.2%). The co-mutation landscape differed between CEBPA subgroups. The CEBPA^bZIP-inf^ group exhibited a higher frequency of GATA2 and WT1 mutations, with a complete absence of NPM1 mutations, while the CEBPA^Other^ group exhibited a higher overall frequency of co-mutations (Figure 4a). Differences were also observed between high and low bZIP-domain VAF groups: the high VAF group had a higher frequency of CSF3R mutations but a lower frequency of GATA2 mutations (Figure 4b).

### 3.3. Impact of CEBPA Molecular Characteristics on Patient Prognosis

**Figure 5:**
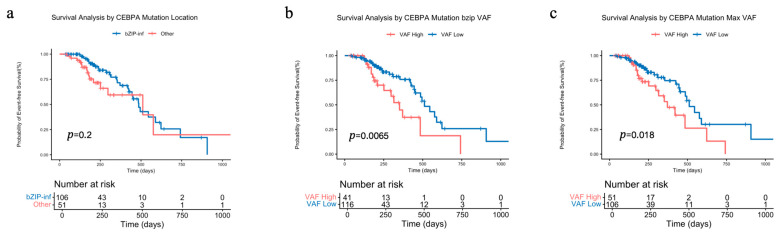
Impact of CEBPA molecular characteristics on event-free survival (EFS): (**a**) EFS analysis stratified by CEBPA mutation type (CEBPA^bZIP-inf^ vs. CEBPA^Other^). (**b**) EFS analysis stratified by CEBPA bZIP-domain VAF. (**c**) EFS analysis stratified by CEBPA maximum VAF.

In the overall cohort of CEBPA-mutated AML patients, we observed no significant difference in EFS between the CEBPA^bZIP-inf^ and CEBPA^Other^ group (*p* = 0.2, Figure 5a). However, stratification by VAF of CEBPA mutation showed a significant prognostic impact. Grouping based on the bZIP-domain VAF provided superior discrimination for EFS (median survival: 351 days vs. 511 days; HR = 3.174, *p* = 0.0065, Figure 5b) compared to grouping by the maximum VAF (median survival: 371 days vs. 511 days; HR = 2.460, *p* = 0.018, Figure 5c).

**Figure 6:**
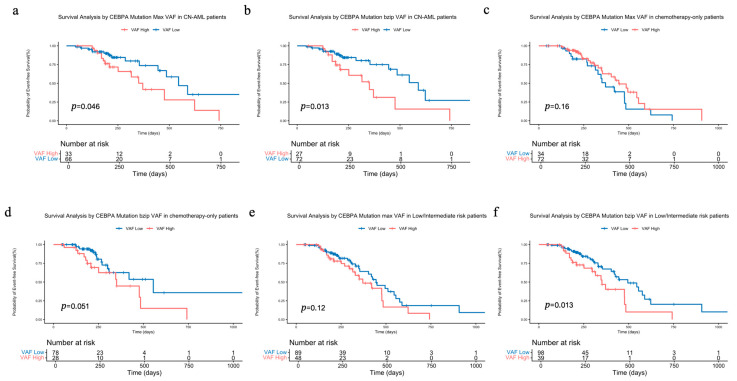
Prognostic value of CEBPA mutation VAF in different AML subgroups. Impact on EFS in CN-AML patients stratified by (**a**) maximum VAF and (**b**) bZIP-domain VAF. Impact on EFS in chemotherapy-only patients stratified by (**c**) maximum VAF and (**d**) bZIP-domain VAF. Impact on EFS in low/intermediate-risk patients stratified by (**e**) maximum VAF and (**f**) bZIP-domain VAF.

Given the demonstrated prognostic utility of CEBPA bZIP-domain VAF, we further evaluated its predictive capacity within different AML subgroups. Analysis focused on the 99 patients with cytogenetically normal AML (CN-AML) revealed that both maximum VAF and bZIP-domain VAF maintained prognostic significance in this subgroup (*p* = 0.046 vs. *p* = 0.013, Figure 6a,b).

Among the 106 patients treated with chemotherapy-only, stratification by maximum VAF showed no significant EFS difference (*p* = 0.16, Figure 6c). However, shorter EFS was observed in the high bZIP-domain VAF group (*p* = 0.051, Figure 6d). We next assessed the prognostic value of CEBPA bZIP-domain VAF in the 137 patients classified as low- or intermediate-risk based on 2022 ELN guidelines, and found bZIP-domain VAF still (*p* = 0.12 vs. *p* = 0.013, Figure 6e,f) effectively stratified patient outcomes.

### 3.4. COX Regression Analysis of Molecular and Clinical Characteristics for Patient Prognosis

Univariate and multivariate COX regression analyses were performed to assess the impact on event-free survival (EFS), incorporating clinical characteristics (age, white blood cell count, hemoglobin level, platelet count, co-occurring molecular mutations, ELN risk stratification, and karyotype) and molecular features of CEBPA mutations (mutation sites and bZIP-domain VAF). The analysis identified the presence of a DNMT3A mutation and a high bZIP-domain VAF as independent risk factors associated with shorter EFS (Table 4).

## 4. Discussion

In this study, we analyzed a Chinese patient cohort to explore the impact of molecular features of CEBPA mutations on clinical outcomes. Our analysis confirmed the specific hotspots of CEBPA mutations and the younger age of CEBPA^bZIP-inf^ patients that were reported in previous studies [15]. Furthermore, we identified higher hemoglobin levels and lower platelet counts in the CEBPA^bZIP-inf^ group, a finding not commonly reported in Western cohorts but consistent with a prior Chinese study [16], suggesting that this may be a characteristic unique to Chinese patients.

The analysis of co-mutations suggests a putative mechanism underlying the prognostic differences. The presence of a higher GATA2 mutation burden in the CEBPA^bZIP-inf^ group may confer a survival advantage [17], whereas the CEBPA^Other^ group’s inferior outcomes are potentially driven by a greater prevalence of high-risk mutations, including FLT3-ITD and ASXL1 [18,19]. This principle extends to VAF-based stratification. The high VAF subgroup’s adverse prognosis may be explained by a higher incidence of CSF3R mutations, suggesting a synergistic leukemogenic effect [20], and a concomitant reduction in GATA2 mutations, thereby attenuating their well-established favorable prognostic influence [21,22].

The seminal studies by Wakita et al., Taube et al., and Georgi et al., utilizing large clinical cohorts, systematically analyzed the prognostic differences of CEBPA mutation types (single/double allele, bZIP/non-bZIP) and established the independent prognostic value of in-frame bZIP-domain mutations, providing key evidence for recent updates in AML risk stratification guidelines [6,10,15]. However, relapse still significantly shortens the survival of CEBPA^bZIP-inf^ patients. Among patients classified as low/intermediate-risk in our cohort, approximately 38.7% (53/137) experienced disease relapse. Notably, even among those who underwent transplantation following the first CR, the relapse rate remained as high as 27.3% (6/22) [23]. Our analysis also suggested that the predictive power of mutation sites alone for the overall CEBPA-mutated population is limited. Potential explanations include the retrospective design, limited sample size, or confounding effects of therapies like transplantation. Despite this, these findings still suggest potential limitations in prognosis assessment based on mutation sites.

In contrast to previous studies, this research focuses more on the role of VAF of CEBPA mutations in patient prognosis stratification. With the advancement and widespread use of NGS, VAF has become an easily accessible and analyzable metric, and its importance cannot be overlooked. We found that the VAF of CEBPA mutations was closely associated with the leukemia burden in patients, suggesting its potential value in predicting prognosis. Given that most patients carry biallelic mutations, presenting with 2–3 VAF values, our study first compared the prognostic utility of using the maximum VAF versus the functionally relevant bZIP-domain VAF as representative values. Using ROC curve analysis and bootstrap resampling, we established a robust VAF threshold of 44.2%, which is closely aligned with the 45.45% cutoff reported in a previous study [13].

In the overall cohort, survival analysis confirmed a higher hazard ratio (HR) for stratification by bZIP-domain VAF, indicating superior risk stratification. This indicates that the prognostic significance of VAF is highly dependent on the functional context of the mutated domain. Furthermore, we demonstrated the robust and significant prognostic value of bZIP-domain VAF in key subgroups, which confirmed it as a stable and reliable predictor of prognosis in various patient populations. Since most patients with CEBPA mutations are classified as low/intermediate-risk and are not initially considered for transplantation, the association of high bZIP-domain VAF with poorer event-free survival and a more unfavorable co-mutation profile in these two subgroups holds significant implications for early clinical decision-making. For patients with high bZIP-domain VAF, we recommend considering earlier implementation of intensive therapeutic strategies, such as intensified chemotherapy or bridging to transplantation, to improve survival outcomes.

Multivariate COX regression analysis incorporating clinical and molecular features identified DNMT3A co-mutation and high bZIP-domain VAF as independent risk factors for inferior EFS. This aligns with previous studies showing poor outcomes for patients with CEBPA/DNMT3A co-mutations [24], suggesting that despite being classified as intermediate risk, these patients may require early intensive therapy or transplantation to improve survival.

However, this study has limitations. First, as a single-center retrospective study, heterogeneity existed in patient treatment regimens. Although the majority received induction therapy based on the “3 + 7” protocol, variations existed in the specific agents used among different patients. Additionally, 45 patients received reduced-intensity chemotherapy, represented by venetoclax plus azacitidine, due to intolerance to intensive chemotherapy. Differences in treatment intensity may influence prognosis, constituting uncontrolled confounding factors. Second, the prognostic model based on bZIP-domain VAF achieved an area under the receiver operating characteristic curve (AUC) of 0.57, indicating that this single variable alone has limited power for precise individual-level prognosis prediction. No single biomarker can achieve perfect risk stratification in a highly heterogeneous disease like AML. The 44% cutoff used herein should be regarded as an exploratory threshold, intended primarily for preliminary subgroup analysis rather than as a universal clinical diagnostic standard. However, based on multivariate analysis and bootstrap validation, the threshold we employed still provides empirical support and a reference for subsequent related analyses. Furthermore, a significant limitation is the difficulty in externally validating the specific mutation sites and their variant allele frequencies (VAFs) in an independent cohort, as the vast majority of public AML datasets do not provide the raw sequencing reads required for VAF calculation. Although the robustness of our findings was rigorously assessed using internal resampling validation, future prospective studies employing uniform sequencing and analytical protocols are warranted to confirm the generalizability of our results. Finally, the median follow-up time in this study remains relatively short, and longer follow-up may help further clarify the impact of VAF on long-term survival outcomes. Meanwhile, the small number of patients who underwent allogeneic hematopoietic stem cell transplantation after first complete remission (24 patients) precludes a robust evaluation of the independent prognostic value of bZIP-domain VAF in the transplanted population within this cohort. Future prospective multicenter studies validating these findings within a unified treatment context and incorporating multi-omics information (e.g., concomitant mutation profiles, minimal residual disease status) will be crucial to establish clinically applicable cutoffs for bZIP-domain VAF and improve its predictive performance.

## 5. Conclusions

In conclusion, based on a large single-center Chinese cohort, this study demonstrates that in patients harboring two or more CEBPA mutations, bZIP-domain VAF is a more powerful prognostic tool than maximum VAF. The core innovation of this work lies in systematically demonstrating that high bZIP-domain VAF defines a distinct subgroup characterized by a high frequency of CSF3R mutations, a low frequency of GATA2 mutations, and significantly inferior event-free survival. Firstly, we provide the first evidence that high bZIP-domain VAF is associated with poor patient prognosis, enabling early identification of high-risk subgroups, and preliminarily propose a potential threshold. Secondly, by comparing bZIP-domain VAF with maximum VAF, we offer practical guidance for selecting the most prognostically relevant metric in clinical practice. Finally, we validate the robust prognostic value of bZIP-domain VAF in patients receiving chemotherapy-only and within the ELN low/intermediate-risk groups, highlighting its potential for early therapeutic decision-making, such as prompting consideration for allogeneic hematopoietic stem cell transplantation. Furthermore, at the mechanistic research level, the potential differences in co-mutation patterns and clonal structures between high- and low-VAF subgroups provide new insights into the biological heterogeneity of CEBPA-mutated leukemia. These differences may reflect distinct clonal evolutionary pathways or selective pressures during leukemogenesis, which could, in turn, be linked to variations in treatment response and the development of drug resistance mechanisms. Therefore, VAF is not only a prognostic marker but may also serve as a critical entry point for exploring disease biology, identifying key synergistic events, and even developing novel combination therapeutic strategies.

Although the optimal VAF threshold still requires further validation in larger patient cohorts with longer follow-up periods, our findings strongly advocate for the incorporation of bZIP-domain VAF into the molecular diagnostic and risk assessment framework for CEBPA-mutated AML. This study thereby advances the field from a categorical understanding of CEBPA mutations toward a more precise, quantitative model for personalized prognosis and treatment stratification.

## Figures and Tables

**Table 1:** Clinical characteristics of patients grouped by CEBPA mutation sites.

	CEBPA^bZIP-inf^	CEBPA^Other^	*p*-Value
n (%)	107 (66.1%)	55 (33.9%)	
Age, y, Median (IQR)	39 (31–53)	54 (42–65)	<0.0001
Sex, n (%)			0.7415
Female	51 (47.7%)	28 (50.9%)	
Male	56 (52.3%)	27 (49.1%)	
FAB, n (%)			0.9999
M2	62 (57.9%)	30 (54.6%)	
Other Type	14 (13.1%)	7 (12.7%)	
Unknown	31 (29.0%)	18 (32.7%)	
Laboratory, Median (IQR)			
BM blasts, %	60.0 (46.0–75.0)	57.6 (39.0–72.0)	0.4331
WBC, ×10^9^/L	13.8 (6.6–46.2)	12.9 (4.4–44.1)	0.5101
HB, g/L	99.0 (82.0–114.0)	80.0 (66.0–100.0)	0.0001
PLT, ×10^9^/L	29.0 (18.0–56.0)	58.0 (22.0–98.0)	0.0027
Cytogenetics, n (%)			0.8328
Normal karyotype	69 (64.5%)	35 (63.6%)	
Aberrant karyotype	20 (18.7%)	11 (20.0%)	
Unknown	18 (16.8%)	9 (16.4%)	
Treatment, n (%)			0.3374
Allo-HSCT	26 (24.3%)	15 (27.3%)	
Chemotherapy only	101 (75.7%)	40 (72.7%)	
CEBPA bZIP VAF, %, Median (IQR)	42.3 (39.6–45.3)	41.8 (30.1–47.0)	0.1647

**Table 2:** Clinical characteristics of patients grouped by CEBPA mutation maximum VAF.

	VAF < 44.2%	VAF > 44.2%	*p*-Value
n (%)	110 (67.9%)	52 (32.1%)	
Age, y, Median (IQR)	45.0 (31.8–60.0)	43.0 (36.3–53.8)	0.6946
Sex, n (%)			0.0413
Female	60 (54.5%)	19 (36.5%)	
Male	50 (45.5%)	33 (63.5%)	
FAB, n (%)			0.1143
M2	67 (60.1%)	25 (48.1%)	
Other	11 (10.0%)	10 (19.2%)	
Unknown	32 (29.9%)	17 (32.7%)	
Laboratory, Median (IQR)			
BM blasts	55.0 (43.6–69.0)	67.3 (47.5–82.5)	0.0091
WBC, ×10^9^/L	9.0 (4.4–20.8)	44.9 (19.2–120.0)	<0.0001
HB, g/L	96.0 (75.8–110.0)	90.0 (73.3–104.5)	0.2129
PLT, ×10^9^/L	39.0 (21.8–74.3)	26.0 (13.3–54.8)	0.0050
Cytogenetics, n (%)			0.5277
Normal karyotype	68 (61.8%)	35 (67.3%)	
Aberrant karyotype	24 (21.8%)	9 (17.3%)	
Unknown	18 (16.4%)	8 (15.4%)	
Treatment, n (%)			0.7235
Allo-HSCT	35 (31.8%)	18 (34.6%)	
Chemotherapy only	75 (68.2%)	34 (65.4%)	
CEBPA Mutation Sites, n (%)			0.7266
CEBPA^bZIP-inf^	73 (66.4%)	33 (63.5%)	
CEBPA^Other^	37 (33.6%)	19 (36.5%)	

**Table 3:** Clinical characteristics of patients grouped by CEBPA mutation bZIP domain VAF.

	VAF < 44.2%	VAF > 44.2%	*p*-Value
n (%)	118 (72.8%)	44 (27.2%)	
Age, y, Median (IQR)	44.5 (32.0–58.3)	43.0 (36.3–58.8)	0.6275
Sex, n (%)			0.1153
Female	62 (52.5%)	17 (38.6%)	
Male	56 (47.5%)	27 (61.4%)	
FAB, n (%)			0.6555
M2	70 (59.3%)	22 (50.0%)	
Other	15 (12.7%)	6 (13.6%)	
Unknown	33 (28.0%)	16 (36.4%)	
Laboratory, Median (IQR)			
BM blasts	54.5 (42.6–67.4)	72.8 (53.9–85.0)	<0.0001
WBC, ×10^9^/L	9.7 (4.5–25.2)	44.9 (17.3–120.0)	<0.0001
HB, g/L	96.5 (75.8–114.3)	87.5 (73.0–102.8)	0.0922
PLT, ×10^9^/L	38.0 (20.0–68.3)	27.5 (15.0–58.0)	0.1078
Cytogenetics, n (%)			0.2942
Normal karyotype	74 (62.7%)	30 (68.2%)	
Aberrant karyotype	25 (21.2%)	6 (13.6%)	
Unknown	19 (16.1%)	8 (18.2%)	
Treatment, n (%)			0.5209
Allo-HSCT	29 (24.6%)	13 (29.5%)	
Chemotherapy only	89 (75.4%)	31 (70.5%)	
CEBPA Mutation Location, n (%)			0.1297
CEBPA^bZIP-inf^	82 (69.5%)	25 (56.8%)	
CEBPA^Other^	36 (30.5%)	19 (43.2%)	

**Table 4:** Univariate and multivariate COX regression analyses of clinical and molecular characteristics in CEBPA-mutated AML patients.

	Univariate Analysis		Multivariate Analysis	
	HR (95%CI)	*p*-Value	HR (95%CI)	*p*-Value
Age (>43 y vs. <43 y)	1.501 (0.829–2.718)	0.180		
WBC × 10^9^/L(<12.7 vs. >12.7)	0.646 (0.355–1.177)	0.153		
Hb (>95 g/Lvs. <95 g/L)	0.685 (0.375–1.251))	0.219		
PLT × 10^9^/L (>35 vs. <35)	0.816 (0.441–1.511)	0.518		
WT1 Mutation (Yes vs. No)	0.752 (0.407–1.390)	0.363		
TET2 Mutation (Yes vs. No)	1.242 (0.618–2.493)	0.543		
GATA2 Mutation (Yes vs. No)	0.724 (0.281–1.866)	0.503		
FLT3-ITD (Yes vs. No)	3.038 (1.439–6.413)	0.004	1.470 (0.627–3.445)	0.375
KIT Mutation (Yes vs. No)	0.965 (0.342–2.723)	0.947		
CSF3R Mutation (Yes vs. No)	1.937 (0.799–4.695)	0.143		
DNMT3A Mutation (Yes vs. No)	8.410 (3.407–20.756)	<0.001	6.275 (2.303–17.097)	<0.001
NRAS Mutation (Yes vs. No)	0.829 (0.255–2.699)	0.756		
Risk Classification (Favor vs. Adver)	1.544 (0.851–2.800)	0.153		
Karyotype (Complex vs. Normal)	2.127 (0.894–5.061)	0.088	1.710 (0.706–4.142)	0.234
Chemotherapy Method (Standard vs. Reduced)	0.935 (0.435–2.008)	0.863		
CEBPA Sites (bZIP-inf vs. Other)	1.506 (0.805–2.819)	0.200		
bZip-domain VAF (High vs. Low)	2.327 (1.246–4.345)	0.008	1.960 (1.034–3.716)	0.039

## Data Availability

The datasets generated during and/or analyzed during the current study are available from the corresponding author on reasonable request.

## Supplementary Materials

The following supporting information can be downloaded at: https://www.mdpi.com/article/10.3390/biomedicines14010256/s1, Table S1: Genes included in the 139-gene myeloid targeted NGS panel; Figure S1: Determination of the optimal cutoff value for CEBPA mutation VAF. (a) Time-dependent ROC curve analysis for the maximum VAF (b) Time-dependent ROC curve analysis for the bZIP-domain VAF; Figure S2: Bootstrap analysis of CEBPA mutation maximum VAF threshold. (a) Distribution of bootstrap replicates for the maximum VAF threshold. (b) Distribution of bootstrap replicates for the AUC values of the maximum VAF threshold. (c) Scatter plot of the joint distribution between the maximum VAF threshold and AUC value bootstrap replicates. (d) Event-free survival analysis based on the optimal threshold (44.2%); Figure S3: Bootstrap analysis of CEBPA mutation bZIP region VAF threshold. (a) Distribution of bootstrap replicates for the bZIP-domain VAF threshold. (b) Distribution of bootstrap replicates for the AUC values of the bZIP-domain VAF threshold. (c) Scatter plot of the joint distribution between bZIP-domain VAF threshold and AUC value bootstrap replicates. (d) Event-free survival analysis based on the optimal threshold (44.2%); Figure S4: Impact of copy number variations on event-free survival. (a) Impact of WT1-associated 11p CN-LOH on EFS. (b) Impact of CEBPA-associated 19q CN-LOH on EFS.

## Abbreviations

The following abbreviations are used in this manuscript:

AML	Acute myeloid leukemia
VAF	Variant allele frequency
CNVs	Copy number variations
NGS	Next-generation sequencing
EFS	Event-free survival
CN-LOH	Copy-neutral loss of heterozygosity
CEBPA	CCAAT/enhancer-binding protein α
bZIP	Basic region leucine zipper
CN-AML	Cytogenetically normal AML
Allo-HSCT	Allogeneic hematopoietic stem cell transplantation
CR	Complete remission
