# Supplementary material for: bZIP-Domain Variant Allele Frequency Helps to Refine Risk Stratification in CEBPA-Mutated AML
Source: Biomedicines. 2026 Jan 22;14(1):256. doi: 10.3390/biomedicines14010256 (PMC12839442; doi:10.3390/biomedicines14010256)
Supplement: Supplementary file 1 [file biomedicines-14-00256-s001.zip › biomedicines-4012737-supplementary.pdf]

Table S1. Genes included in the 139-gene myeloid targeted NGS panel.

ABCB1	BRAF	CTCF	FLT3	JAK1	NRAS	SETBP1	TCF3
ABL1	BRCA1	CUX1	G6PC3	JAK2	NT5C2	SETD2	TERC
AKT3	BRCA2	DDX41	GATA1	JAK3	NTRK1	SETDB1	TERT
ANKRD26	BRIP1	DIS3	GATA2	KDM6A	PAX5	SF1	TET1
ARID1A	CALR	DKC1	GATA3	KIT	PDGFRA	SF3A1	TET2
ARID1B	CBL	DNMT3A	GFI1	KMT2A	PDGFRB	SF3B1	TP53
ARID2	CBLB	EED	GNAS	KMT2B	PHF6	SH2B3	TPMT
ASXL1	CBLC	EGFR	GNB1	KMT2C	PIGA	SMC1A	U2AF1
ASXL2	CCND3	EGLN1	GSKIP	KMT2D	PML	SMC3	U2AF2
ATG2B	CDKN1A	ELANE	HAX1	KRAS	PPM1D	SOCS1	VHL
ATM	CDKN2A	EP300	HRAS	LMO2	PRPF8	SRP72	WAS
ATRX	CDKN2B	EPOR	ID3	MPL	PTEN	SRSF2	WT1
BCL2	CEBPA	ERG	IDH1	MYC	PTPN11	STAG2	ZRSR2
BCL6	CHEK2	ETNK1	IDH2	NBN	RAD21	STAT3	
BCOR	CREBBP	ETV6	IKZF1	NF1	RARA	STAT5A	
BCORL1	CRLF2	EZH2	IKZF2	NOTCH1	RB1	STAT5B	
BLM	CSF1R	FBXW7	IKZF3	NOTCH2	RUNX1	SUZ12	
BPGM	CSF3R	FGFR3	IL7R	NPM1	SBDS	TAL1	

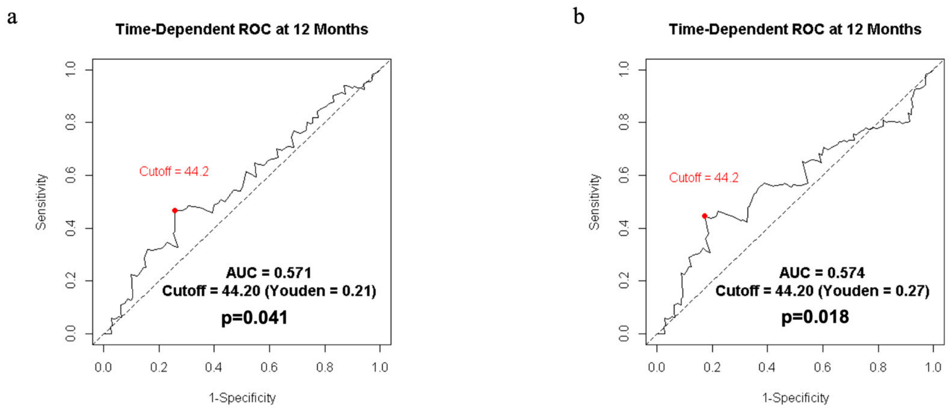

Figure S1. Determination of the optimal cut-off value for CEBPA mutation VAF.(a) Time-dependent ROC curve analysis for the maximum VAF (b). Time-dependent ROC curve analysis for the bZIP-domain VAF.

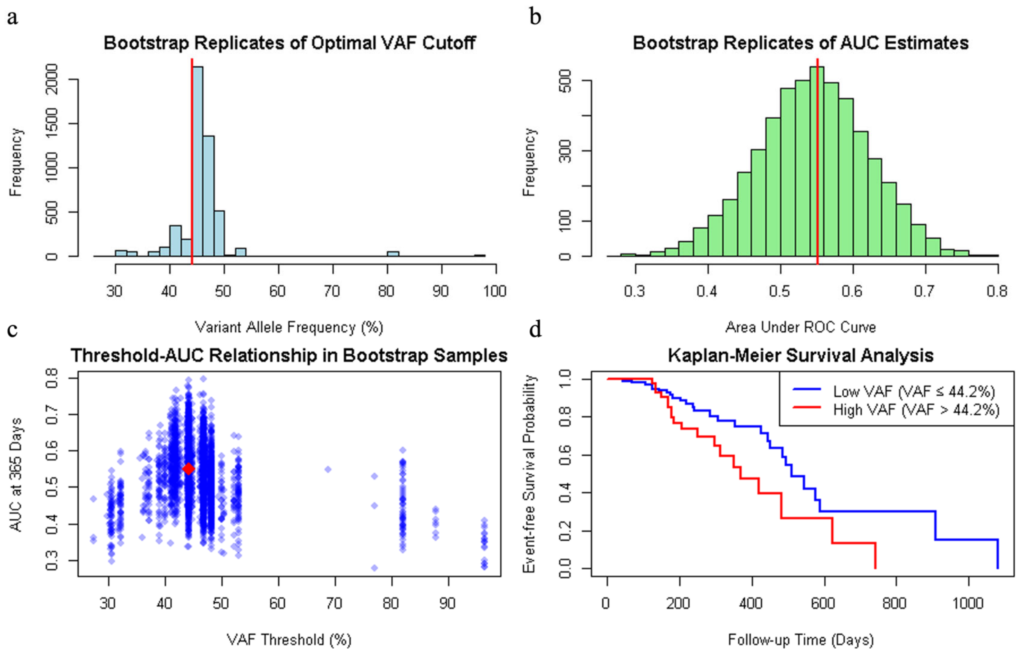

Figure S2. Bootstrap analysis of CEBPA mutation maximum VAF threshold. (a) Distribution of bootstrap replicates for the maximum VAF threshold (b) Distribution of bootstrap replicates for the AUC values of the maximum VAF threshold (c) Scatter plot of the joint distribution between the maximum VAF threshold and AUC value bootstrap replicates (d) Event-free Survival analysis based on the optimal threshold (44.2%)

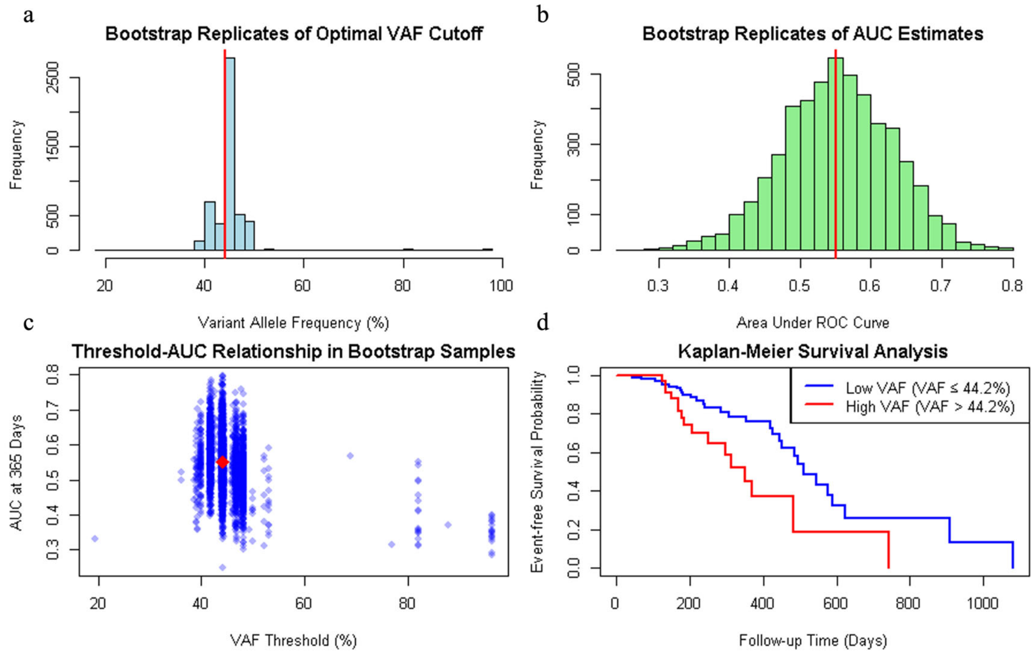

Figure S3. Bootstrap analysis of CEBPA mutation bZIP region VAF threshold. (a) Distribution of bootstrap

replicates for the bZIP-domain VAF threshold (b) Distribution of bootstrap replicates for the AUC values of the bZIP-domain VAF threshold (c) Scatter plot of the joint distribution between bZIP-domain VAF threshold and AUC value bootstrap replicates (d) Event-free Survival analysis based on the optimal threshold (44.2%)

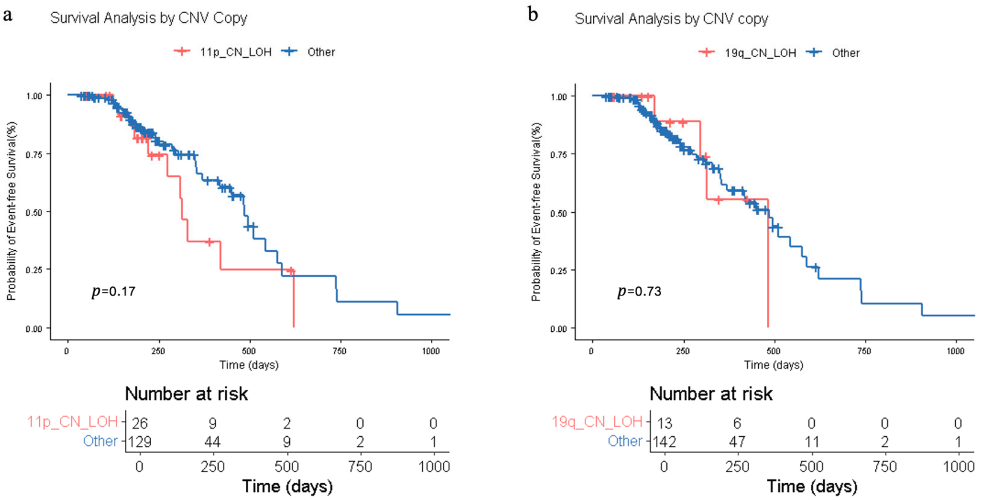

Figure S4. Impact of copy number variations on event-free survival. (A) Impact of *WT1*-associated 11p CN-LOH on EFS. (B) Impact of *CEBPA*-associated 19q CN-LOH on EFS.
